# A placebo-controlled, crossover trial to investigate the efficacy of tiotropium bromide or placebo added to usual care in stable symptomatic post-hematopoietic stem cell transplantation (HSCT) bronchiolitis obliterans syndrome (BOS)

**DOI:** 10.1186/s13063-024-08051-7

**Published:** 2024-04-06

**Authors:** Naeemeh Dini, Amin Pastaki Khoshbin, Rasoul Aliannejad, Hooman Bakhshandeh, Katayoun Najafizadeh, Mahshid Mehdizadeh, Shahideh Amini

**Affiliations:** 1 Department of Clinical Pharmacy, School of Pharmacy, Tehran University of Medical Sciences, Tehran, Iran; 2 School of Medicine, Tehran University of Medical Sciences, Tehran, Iran; 3 Thoracic Research Center, Department of Internal Medicine, Division of Pulmonary and Critical Care, Shariati Hospital, Tehran University of Medical Sciences, Tehran, Iran; 4 Rajaie Cardiovascular Medical and Research Center, Iran University of Medical Sciences, Tehran, Iran; 5 Shahid Beheshti University of Medical Sciences, Tehran, Iran; 6 Hematopoietic Stem Cell Research Center, Shahid Beheshti University of Medical Sciences, Tehran, Iran

**Keywords:** Bronchiolitis obliterans, Obstructive lung function, Tiotropium

## Abstract

**Background:**

Despite the fundamental progress in hematopoietic stem cell transplant, this treatment is also associated with complications. Graft-versus-host disease is a possible complication of HSCT. Bronchiolitis obliterans syndrome (BOS) is the pulmonary form of this syndrome. Due to the high morbidity and mortality rate of BOS, various studies have been conducted in the field of drug therapy for this syndrome, although no standard treatment has yet been proposed. According to the hypotheses about the similarities between BOS and chronic obstructive pulmonary disease, the idea of using tiotropium bromide as a bronchodilator has been proposed.

**Method/design:**

A randomized, double-blind, placebo-controlled, and crossover clinical trial is being conducted to evaluate the efficacy of tiotropium in patients with BOS. A total of 20 patients with BOS were randomly assigned (1:1) to receive a once-daily inhaled capsule of either tiotropium bromide (KP-Tiova Rotacaps 18 mcg, Cipla, India) or placebo for 1 month. Patients will receive tiotropium bromide or placebo Revolizer added to usual standard care. Measurements will include spirometry and a 6-min walking test.

**Ethics/dissemination:**

This study was approved by the Research Ethics Committees of Imam Khomeini Hospital Complex, Tehran University of Medical Science. Recruitment started in September 2022, with 20 patients randomized. The treatment follow-up of participants with tiotropium is currently ongoing and is due to finish in April 2024. The authors will disseminate the findings in peer-reviewed publications, conferences, and seminar presentations.

**Trial registration:**

Iranian Registry of Clinical Trial (IRCT) IRCT20200415047080N3. Registered on 2022–07-12, 1401/04/21.

**Supplementary Information:**

The online version contains supplementary material available at 10.1186/s13063-024-08051-7.

## Background

Pulmonary complications account for more than 90% of mortality after bone marrow transplant (BMT). Bronchiolitis obliterans syndrome (BOS) is one of them, which is defined as the pulmonary form of graft-versus-host disease (GVHD). Due to post-bone marrow transplant bronchiolitis obliterans syndrome (PBMTBOS), high morbidity and mortality rate, various studies have been conducted in the field of drug therapy [1], although no standard treatment has yet been proposed [2, 3].

Different treatments have been investigated for this disease, but if new bronchiolitis obliterans have been diagnosed, azithromycin, along with immunosuppressant and adjustment of the immunosuppressant regimen, is one of the therapeutic strategies [2–9].

Systemic corticosteroids, along with other immunosuppressive drugs, are known as the core of treatment in BOS [2]. Clinical studies conducted so far have not seen a significant effect on the treatment of patients by adding azathioprine, mycophenolate, thalidomide, and hydroxychloroquine [10–12]. On the other hand, a 2002 study by Koc et al. concluded the use of cyclosporine as corticosteroid-sparing can be considered [13].

Bergeron et al., in 2014, in a randomized، double-blind clinical trial on PBMTBOS, evaluated the possible effect of budesonide/formoterol in these patients. In this study, the FEV1 saw an increase in the treatment group [14].

In another study conducted in 2005 by Khalid et al., the effect of azithromycin on PBMTBOS was evaluated. Based on the results of this study, patients showed an increase in forced vital capacity and FEV1 compared to baseline [15]. Also, in another study conducted by Vos et al., in post lung transplant BOS (PLTBOS), the results showed that long-term use of azithromycin can reduce the rate of drop in FEV1 in patients [16].

Williams and colleagues evaluated the effect of fluticasone/azithromycin/montelukast regimens in a 2016 study on patients with PBMTBOS. The study reported significant improvements in FEV1, functional status, and life satisfaction in patients [17].

Due to the different possible processes in disease pathogenesis, several studies have investigated the role of TNF-alpha inhibitors as well as drugs such as imatinib and ruxolitinib [2]. In a study conducted by Zeiser et al. on 41 patients with chronic GVHD on the efficacy of ruxolitinib, the results of the study showed an improvement in respiratory symptoms in 4 patients with respiratory GVHD in this study [18]. In another study by Olivieri and colleagues on the possible benefits of imatinib in the treatment of 19 patients with chronic GVHD, 7 out of 11 patients with pulmonary involvement showed improvement in respiratory function [19].

Few studies evaluated the effect of tiotropium in the treatment of BOS. In 2014, Kawassaki and colleagues investigated the possible impact of tiotropium bromide on the treatment of patients with secondary constrictive bronchiolitis due to various causes. In this study, 11 patients with constrictive bronchiolitis were treated with tiotropium bromide for an average of 21 days (with a minimum treatment period of 14 days), and spirometry tests were evaluated before and after treatment in the patients. According to the results of this study, FEV1 saw a rise. Also, FVC levels increased, although no significant difference was observed in FEV1/FVC levels. This study was conducted without a control group, and the results were only compared before and after the intervention [20].

Bronchiolitis obliterans (BO) is believed to be caused by a myriad of reasons like infection, post-toxin exposure, post HSCT and post lung transplant. Following the data, however, somehow in PLTBOS, fibrosis might be made and the treatment might be different. But generally speaking, BO refers to the small-airway epithelial cells and subepithelial structure changes which cause excessive fibroproliferation due to aberrant tissue repair. The disease is mentioned to cause air trapping, mosaic attenuation, and hyperinflation and is defined as an obstructive pulmonary function. The disease is pure obliteration of the small airways (< 2 mm) [5, 21].

All in all, for patients with bronchiolitis obliterans who have undergone HSCT or lung transplantation, several immunosuppressive medications and immune-modulating treatments have been reported to stabilize pulmonary function in BOS patients in some trials. Low-dose macrolide antibiotics (azithromycin), leukotriene-receptor antagonists (montelukast), and combinations of inhaled bronchodilators and glucocorticoids are believed to help BOS symptoms and halt the decrease in pulmonary function tests [21], but more studies are needed to find the best protocol for disease management.

Tiotropium is an anticholinergic drug that is used in the management of chronic obstructive pulmonary disease (COPD) and is believed to have anti-inflammatory effects as well [22]. It has been studied in post-infectious and post-HSCT BOS and a decrease in airway obstruction and improvement in lung function was reported [23, 24]. According to the hypotheses about the similarities between BOS and COPD, the idea of using tiotropium bromide as a bronchodilator has been proposed. This is to observe and evaluate the changes of % FEV1 with the tiotropium add-on therapy.

## Methods/design

### Study design

**Fig. 1:**
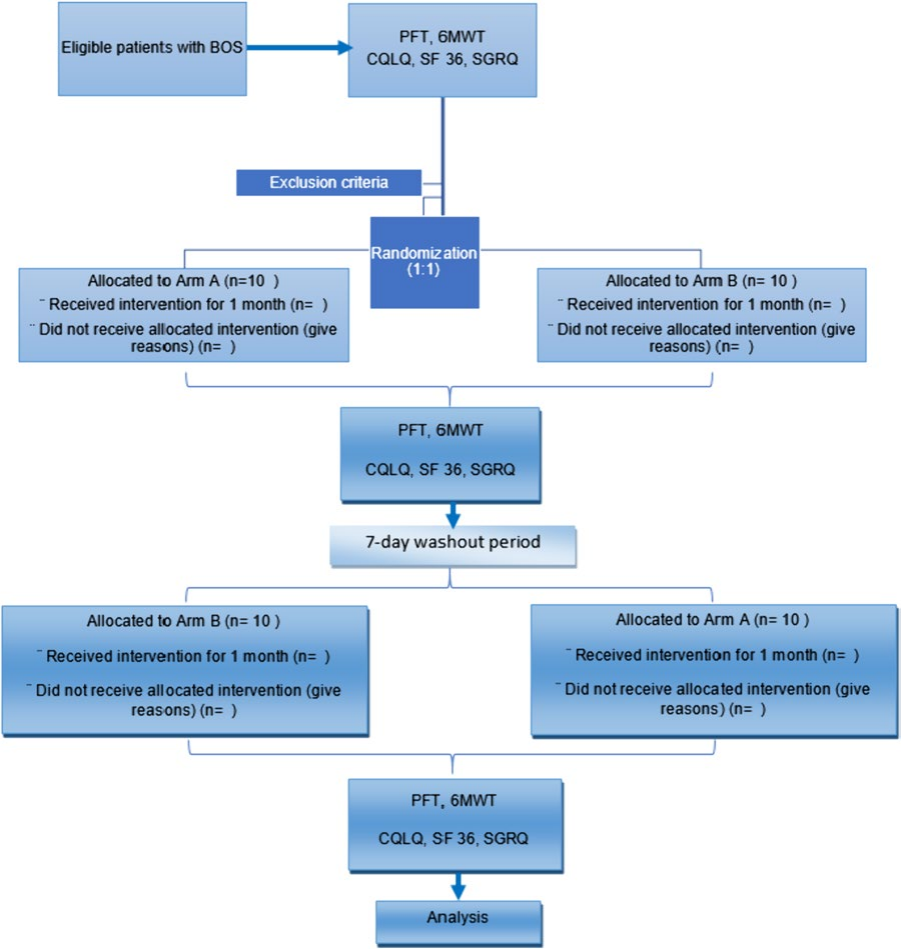
Study design overview

This is a randomized, double-blind, placebo-controlled, crossover trial of maintenance treatment with once-daily tiotropium for patients with BOS. The study will be conducted in two general educational hospitals. This paper presents the design and protocol for the trial according to the Standard Protocol Items: Recommendations for Interventional Trials (SPIRIT) statement (Supplementary Material 1) [25]. An overview of the study design and timeline for participants is provided in Fig. 1.

The crossover design will consist of two sequences with two treatment periods (AB/BA or BA/AB) each lasting 1 month and separated by a 1-week wash-out period. In treatment period A or B, patients will receive tiotropium bromide or a placebo Revolizer added to usual standard care. Patients will be screened for eligibility and, once confirmed, will be randomized (1:1) to each treatment sequence. After completing the first treatment period in the sequence, patients will undergo a 1-week wash-out period in which they receive only the usual standard care. Then, they will receive the alternative treatment period which they have not received yet. Patients allocated to treatment sequence 1 will receive treatment period A, then wash-out period, and then treatment period B; patients randomized to treatment sequence 2 will receive treatment period B, then wash-out period, and then treatment period A.

Patients who are referred to pulmonary clinics will be screened for eligibility criteria. Baseline data must be collected before enrollment. Baseline data include age, gender, time from transplantation, time from diagnosis of BOS, prior and current immunosuppressive and topical respiratory medication history, other medications, NIH lung symptom score, and chronic GVHD activity in other organs. Spirometry and a 6-min walking test (6MWT) must be performed before enrollment. All patients are required to complete a 36-Item Short Form Survey (SF-36), Cough Quality-of-Life Questionnaire (CQLQ), and St. George’s Respiratory Questionnaire (SGRQ) before enrollment.

A total of 20 eligible patients will be enrolled in the trial. Central computerized permuted blocked randomization will be performed using a 1:1 allocation schedule with random block sizes of 4. The randomization process is performed by investigators who are neither involved in participant recruitment, nor study conduct and assessment evaluation. The allocation sequence will be concealed from the researchers using sequentially numbered, opaque, and sealed envelopes.

During the treatment period A, patients will receive inhaled tiotropium bromide (KP-Tiova) at a dose of 18 mcg once daily in the morning plus usual standard care. Standard usual care consists of inhaled corticosteroids and long-acting beta-2 agonists in a fixed combination in the formulation and dosage which were prescribed to the patient before study enrollment. Other immunosuppressive or GVHD modifying agents must be administered through the study at a dose similar to the study onset. All the patients must be instructed regarding the self-administration of study treatment at the onset of the trial.

During the treatment period B, patients will receive a tiotropium bromide placebo Revolizer plus standard usual care. The placebo Revolizer is identical to the experimental Revolizer in appearance, smell, taste, and texture and is administered on the same schedule as the experimental treatment.

In the wash-out period, patients receive only standard usual care as above. The wash-out period is supposed to last 1 week.

Patients will continue to receive study schedules until completion of the trial, occurrence of unacceptable adverse events, progressive pulmonary disease, change in immunosuppressive agents due to alterations in GVHD activity in other organs, malignancy relapse or graft failure, and withdrawal of consent.

Assessments will be performed at the end of the first treatment period, at the end of the wash-out period, and at the end of the second treatment period. Assessments include spirometry, 6MWT, NIH lung symptom score, GVHD activity in other organs, SF-36 score, SGRQ score, CQLQ score, VAS score, and monitoring of adverse events.

### Eligible participants

The outpatient BOS cases of the pulmonology clinic of Shariati Hospital and Taleghani Hospital were selected. Patients to be enrolled are required to meet all inclusion criteria listed in Table 1 and not meet any of the exclusion criteria. Those with BOS patients with baseline 20% ≤ %FEV1 < 70% will be selected and contacted by telephone. The eligible participants will be asked to visit the research center.

**Table 1:** Inclusion and exclusion criteria

Exclusion criteria	Inclusion criteria
1) Life expectancy < 6 months at the time of enrollment as judged by the enrolling investigator	1) Subjects able to sign the Informed Consent
2) The need for chronic oxygen therapy	2) 18 years of age or older
3) Baseline %FEV1 ≤ 20%	3) Diagnosis of post-HSCT-BOS according to NIH 2014 criteria
4) History of thoracic air leak syndrome	4) Baseline %FEV1 < 70%
5) Documented respiratory infection	5) Must be symptomatic, defined as NIH Lung Score 1–3, and no other contributing etiology (including cardiac diseases, infection, anemia, or extrinsic thoracic compression) is present for respiratory symptoms
6) Active malignancy	6) Stable disease defined as less than 10% change in %FEV1 during the past 3 months while having no change in immunosuppressive and topical respiratory medications in the past 3 months
7) Graft failure	7) Must have received a combination of inhaled corticosteroid and inhaled long-acting beta-2 agonist during the past 3 months
8) Known history of asthma or chronic obstructive lung disease (COPD)	8) No new or supplemental immunosuppressive therapy for extra-thoracic GVHD during the past 3 months
9) Active smoking during the past 12 months	9) Patients with prior exposure to short-acting or long-acting inhaled anti-cholinergic drugs are eligible unless they have been received within the past 3 months
10) Substance abuse or uncontrolled psychiatric disorder	10) Ability to use Revolizer
11) Pregnant or nursing women	
12) Daily corticosteroid of more than 1 mg/kg prednisone equivalent	
13) Known intolerance or allergy to anticholinergic drugs	
14) History of urinary retention, angle-closure glaucoma, CrCl ≤ 30 ml/min	
15) History of arrhythmia in past years, MI in past 6 months, or hospital admission due to heart failure in past year	
16) Ongoing participation in any other clinical trial (more typical would be using an investigational agent within 28 days of enrollment)	
17) Any condition that, in the opinion of the enrolling investigator, would interfere with the subject's ability to comply with the study requirements	
18) Inability to perform pulmonary function tests (PFT) reliably, as determined by the enrolling investigator or PFT lab	
19) No compliance	

### Follow-up and duration of the study

There will be 4 visits, one baseline, one after 1 month of being included, another one after passing the washout period which is the time of being included in the next period, and the last one is going to be 1 month after. An overview of the measures used at each of the four time points is illustrated in Table 2. To improve the compliance of patients, the strategies are as follows: adhere to the principle of voluntary participation and explain the benefits of this study to the participants and the importance of treatment and adherence to the treatment. The health education process could increase the related knowledge, change the behavior, and enhance the compliance of the patient, but they were excluded if they did not have enough adherence and did not use more than 25% of their drug in the previous period. If a patient chooses not to complete trials, they will participate routinely in pulmonary clinics and will directly be monitored by our pulmonologists they will be excluded from the trial. Tiotropium is a routine drug that is prescribed; therefore, it has known side effects and our pulmonologists are familiar with them. Written informed consent will be obtained from each patient before they begin the trial. All participants will fill in informed consent.

**Table 2:**
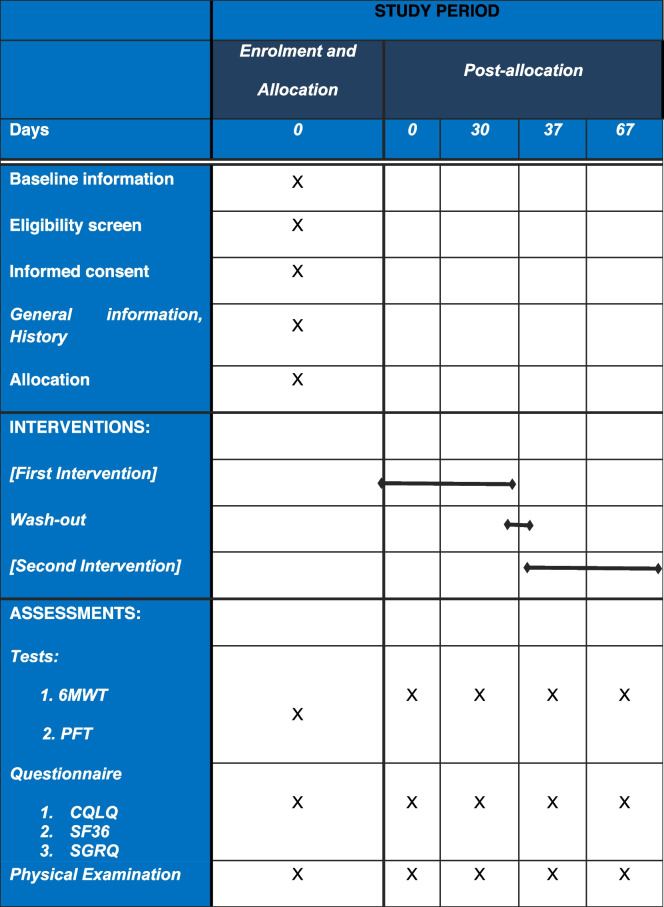
Schedule of enrolment, interventions, and assessments

### Sample size

The sample size calculation for this trial was performed using the PASS 15 [Power Analysis and Sample Size Software (2017). NCSS, LLC. Kaysville, UT, USA]. The primary endpoint of the main study was the surge in %FEV1, a rise from 45% ± 13% to 47% ± 14.5% was seen in the study of Jeong Uk Lim [23], which means a 2% increase in the intervention group. Considering no changes in %FEV1 in the control group, based on a power of 80% and an alpha value of 5% and using the *t*-test for the difference between two means in a 2 × 2 cross-over design, it was calculated that a total sample size of 20 patients (10 in each group) would be sufficient to detect the presumed difference. No drop-out was considered.

### Randomization process

A total of 20 eligible patients will be enrolled in the trial. Central computerized permuted blocked randomization will be performed using a 1:1 allocation schedule with random block sizes of 4. The randomization process is performed by investigators who are not involved in participant recruitment. The allocation sequence will be concealed from the researchers using sequentially numbered, opaque, and sealed envelopes.

### Study drug

Patients will receive inhaled tiotropium bromide (KP-Tiova Rotacaps 18 mcg, Cipla, India) or placebo (KP-Tiova Placebo with lactulose, Cipla, India) which was produced by the same manufacturer. Tiotropium bromide Revolizer or placebo is added to usual standard care and in the treatment period. Standard usual care consists of inhaled corticosteroids and long-acting beta-2 agonists in a fixed combination in the formulation and dosage which were prescribed to the patient before study enrollment. Other immunosuppressive or GVHD modifying agents must be administered through the study at a dose similar to the study onset. All the patients must be instructed regarding the self-administration of study treatment at the onset of the trial. Subjects will be advised to take the study drug at the same time every day according to approved instructions.

### Outcomes

#### Primary outcome

The change from baseline in the measured % FEV1 1 month after treatment with tiotropium bromide Revolizer or placebo added to usual care in stable symptomatic post-HSCT-BOS.

#### Secondary outcomes

1. The change from baseline in the mean forced expiratory volume in 1 s (FEV1) (mL) 1 month after treatment with tiotropium bromide Revolizer or placebo added to usual care in stable symptomatic post-HSCT-BOS.
2. The changes from baseline in the mean percent predicted FEV1 (%FEV1) (%), and FVC after treatment with tiotropium bromide Revolizer or placebo added to usual care in stable symptomatic post-HSCT-BOS
3. The change from baseline in the mean NIH Lung Symptom Score after treatment with tiotropium bromide Revolizer or placebo added to usual care in stable symptomatic post-HSCT-BOS
4. The proportion of complete or partial response rate after treatment with tiotropium bromide Revolizer or placebo added to usual care in stable symptomatic post-HSCT-BOS (in regards to NIH 2014 criteria, a complete response (CR) is defined as normalization of %FEV1 to above 70%. Partial response (PR) is defined as an increase by 10% predicted absolute value of %FEV1)
5. The change from baseline in 6-min walk test distance after treatment with tiotropium bromide Revolizer or placebo added to usual care in stable symptomatic post-HSCT-BOS
6. The change from baseline in SF-36 score after treatment with tiotropium bromide Revolizer or placebo added to usual care in stable symptomatic post-HSCT-BOS
7. The change from baseline in SGRQ score after treatment with tiotropium bromide Revolizer or placebo added to usual care in stable symptomatic post-HSCT-BOS
8. The change from baseline in CQLQ score after treatment with tiotropium bromide Revolizer or placebo added to usual care in stable symptomatic post-HSCT-BOS
9. The change from baseline in VAS score after treatment with tiotropium bromide Revolizer or placebo added to usual care in stable symptomatic post-HSCT-BOS
10. The change in admission rates due to respiratory, mediastinal, and thoracic disease after treatment with tiotropium bromide Revolizer or placebo added to usual care in stable symptomatic post-HSCT-BOS
11. The rate of adverse events rates after treatment with tiotropium bromide Revolizer or placebo added to usual care in stable symptomatic post-HSCT-BOS.

### Adverse events

The adverse events will be examined by open questions, as well as symptoms, and by precise questions about the potentially related adverse effects of the tiotropium which was used in the trial, as xerostomia, pharyngitis, and cardiovascular events like hypertension, edema, and chest pain, etc. The rate of adverse effects will be determined by linking the frequency of adverse events in the experimental drug and placebo by calculating the NARAJNJO scale***.***

### Data entry and management of data files

Data will be entered into a computerized database with the Excel software. All participants’ data and patient health information will be confidential and protected during and after the trial. A code will be used to identify study participants, that will not be given to anybody outside of the study staff except when required by law. Records will be stored in an area with two locked doors. Only the study staff will have access to the locks.

### Monitoring

This is a small investigator-initiated trial, within an academic environment, the responsibility of auditing the trial lies in the hands of the both steering committee and the ethical committee, in which both will have periodical inspections. Details of adverse events will be collected at each study visit. serious adverse events will be reported and assessed by the investigators by means of survival analysis and calculation of hazard ratio.

### Ethics approval and dissemination

Participants will be asked to sign the approved informed consent form before participating in the study. The study protocol (protocol version 1.0, issue date: Dec 2021) was approved by the Ethics Committee of the Imam Khomeini Hospital Complex, Tehran University of Medical Science under the number IR.TUMS.IKHC.REC.1400.361. The trial is conducted in keeping with Good Clinical Practice Guidelines, the ethics drawn in the Declaration of Helsinki, and applicable local laws and regulations. The trial is registered at www.irct.ir under the identification number IRCT20200415047080N3. Informed consent will be signed. Clinical results will be available in a medical journal and presented at national or international conferences. De-identified data can be supplied upon request with data usage agreements to ensure scientific dissemination and transparency. There is no anticipated harm and compensation for trial participation.

### Statistical methods

The analysis will be done by intention-to-treat and per-protocol. Assessment for various effects in the cross-over design will be performed via the following statistical methods:

- Treatment effect (comparison between the intervention and control) will be investigated by paired *t*-test.
- Period effect (the effect of the same treatment received at two different periods is different for each period) will be assessed by the *t* statistics, as follows:
  $$t=\frac{ {dY}_{\mathrm{diff }\ \left(T-P\right)}- {dY}_{{\mathrm{diff}}\ \left(P-T\right)}}{\sqrt{^{{{\text{SD}}}_{\left(T-P\right)}^{2}}\!\left/ \!_{ {n}_{(T-P)}}\right.+^{ {{\text{SD}}}_{\left(P-T\right)}^{2}}\!\left/ \!_{ {n}_{(P-T)}}\right.}}$$
  where *dY*_diff(*T*–*P*)_ is the mean difference in changes of “endpoint Y” in treatment sequence followed by placebo, SD^2^_(*T*–*P*)_ was the variance of the difference of “endpoint Y” and *n*_(*T*–*P*)_ was the number of patients in this sequence. Definitions for the *P*–*T* sequence are the same.
- Period-by-treatment interaction (whether the two treatment effects are different in the two periods), will be evaluated by the t statistic as follows:
  $$t=\frac{ {\mathrm{total}\ \mathrm{Mean}\ dY}_{ \left(T-P\right)}- {\mathrm{total}\ \mathrm{Mean}\ dY}_{ \left(P-T\right)}}{\sqrt{^{{{\text{SD}}}_{\mathrm{total}\ \mathrm{Mean}\ \left(T-P\right)}^{2}}\!\left/ \!_{ {n}_{(T-P)}}\right.+^{ {{\text{SD}}}_{\mathrm{total}\ \mathrm{Mean}\ \left(P-T\right)}^{2}}\!\left/ \!_{ {n}_{(P-T)}}\right.}}$$
  In which:
  $${\mathrm{total}\ \mathrm{Mean}\ dY }_{(T-P)}= \frac{\sum_{1}^{n}{\mathrm{individual\ Mean}\ dY}_{(T-P)}}{{n}_{(T-P)}}$$
  And:
  $${\mathrm{individual\ Mean}\ dY}_{(T-P)}=\frac{{dY}_{\mathrm{ after\ Treatment\ in}\ \left(T-P\right)}+{dY}_{ \mathrm{after\ Placebo\ in}\ \left(T-P\right)}}{2}$$
  It may be considered as the carry-over effect.
- Sequence effect (comparison between the results in AB and BA sequences) will be performed by independent sample *t*-test [26].

As a result of a small sample, no further additional analyses (e.g., subgroup and adjusted analyses) will be done.

## Discussion

Increasing data shows a negligible effect of some immunosuppressants in the pulmonary function of BOS patients; also, there is little available evidence on the impact of inhalation medication influence on the prognosis and the relief of lung function decline. As far as we considered, this study will be the first crossover trial to investigate the efficacy of tiotropium bromide in stable symptomatic post-HSCT, BOS adult patients.

We are all aware of the effect of HSCT on the lives of cancer patients but this comes with indisputable side effects on the lungs, like infection, pulmonary edema, and of course pulmonary GVHD. Generally, GVHD lies in two categories: acute and chronic. The pulmonary GVHD which is a form of chronic GVHD, is called bronchiolitis obliterans and is diagnosed with lung biopsy. The term bronchiolitis obliterans syndrome (BOS) is used when a patient has airflow limitation in the absence of other etiologies, but histopathology to document BO is not available [1–3, 21].

Currently, there is no precise treatment protocol for BOS [1–12, 22]. Based on, Kirsten M. Williams et al.’s study, inhaled fluticasone, azithromycin, and montelukast (FAM) and steroid pulse may halt pulmonary deterioration in new-onset BOS in the majority of patients, therefore the steroid dose could be reduced [17].

As steroids and other immunosuppressants have long-term adverse effects, we believe that with the somehow same pathophysiology of COPD and BOS and the previous study of the tiotropium, it could be a promising drug and may become a great hand.

Little is known about the tiotropium effect on BOS. Jeong Uk Lim et al. disclosed that inhaled tiotropium add-on to a combination of budesonide/formoterol can significantly improve lung function, but not respiratory symptoms, in patients with post-HSCT BOS [23].

Mariângela F C Teixeira et al. conducted a randomized, double-blind, placebo-controlled, crossover, prospective study in patients with stable PIBO, 6 to 16 years of age. They concluded tiotropium acutely decreased airway obstruction and air trapping for up to 24 h in children with postinfectious bronchiolitis obliterans [24].

This study is designed to determine tiotropium effects on lung function in patients with BOS. We hypothesize that tiotropium significantly will improve the FEV1 and symptoms of BOS patients.

A limitation of this study is the sample size is not large enough, and the follow-up time is 1 month. Another limitation is the heterogenicity of bronchodilators and immunosuppressants used by patients.

In summary, our RCT will afford data for analyses of the relationship between BOS and tiotropium, which may be a potential treatment for BOS.

## Trial status

At the time of trial protocol submission, the enrollment of volunteers is ongoing. The study has been ongoing since September 2022. The treatment follow-up of participants with Tiotropium is currently ongoing and is due to finish in April 2024. The protocol version is 1.0.

### Supplementary Information

**Supplementary Material 1.**

## Data Availability

The datasets analyzed during the current study and statistical code are available from the corresponding author on reasonable request, as is the full protocol.
